# Rhinolith or Nasal Calculus*Read before the Wayne County Medical Society, February 17th, 1898.

**Published:** 1898-10

**Authors:** William H. Poole

**Affiliations:** Detroit


### RHINOLITH OR NASAL CALCULUS.*

By William H. Poole, M. D., Detroit.

Mr. President and Members of the Wayne County Medical Society:

The pathological specimen I have the pleasure of exhibiting to you this evening is one of unusual interest, even to those of us who limit our practice to diseases of the eye, ear, nose, and throat, from the infrequency with which we meet these cases, and also from the circumstances which led up to its discovery, owing to the fact that it was situated somewhat differently from most cases of this kind.

Miss L. K., aged twenty-four years, from whose nose this was taken, consulted me January 1st, 1898, regarding her nasal catarrh, with which she stated she had been afflicted ever since

---

* Read before the Wayne County Medical Society, February 17th, 1898.

her childhood. Ten years ago she had been treated for about a year by one of the leading rhinologists of this city, receiving considerable benefit, but for the last two or three years she has had a rather profuse nasal discharge, thickened, and increasingly offensive in character, with obstruction to nasal respiration, loss of smell, nasal voice, and the other usual symptoms which we find in an aggravated case of chronic rhinitis. Lately she had suffered from headache, which was increasing in severity, and was also troubled with weeping of the left eye. She had been using an atomizer for some years without getting any other relief than the keeping of the nose approximately clean.

On making anterior and posterior rhinoscopic examination I found considerable hypertrophy of the turbinates of the left side, especially of the inferior turbinal.

I suggested an operation for the removal of the hypertrophied tissue of the lower turbinal, which was impinging on the floor of the nose. This was agreed upon, and on Saturday, January 15th, I operated at 3 P. M. in the usual way, cocainizing the parts thoroughly and making a practically painless operation.

Hemorrhage was not very profuse, and was readily controlled at this time. The patient returned home, and soon after suffered from an attack of nervous sick headache, to which she was subject upon occasions of nervous strain.

As usual, the headache ended with an attack of retching, after which straining the hemorrhage started in afresh and rather profusely. I tried again to control it with stypics and plugging the naris with absorbent cotton, but did not succeed in thoroughly arresting the flow of blood, and, as the patient was getting very weak, with the kind assistance of Dr. Suttie, I tamponed through the posterior naris with a sponge tent, which instantly stopped the hemorrhage. I then ordered her to be liberally supplied with beef extract, for the double purpose of nourishment and to increase the arterial tension.

Sunday, the next day, she was doing nicely, but was very weak; there was no recurrence of the hemorrhage, but I did not think it advisable to remove the tampon, as she was too weak to bear it.

Monday, January 17th, the patient was a little stronger, but owing to debility I could only remove a part of the tampon from the anterior naris.

The next two days I removed still more of the sponge anteriorly, in all about two-thirds of it being removed up to this time, the patient still being too weak to bear much manipulation.

On Thursday morning, January 20th, I attempted to remove the remainder posteriorly, but found it so firmly fixed that it could not be dislodged except with extreme force under anæsthesia. I called in Dr. Chittick and anæsthetized the patient, when, with considerable difficulty, we removed the remainder of the sponge.

After the patient recovered from the anæsthetic I cleansed the nasal cavity thoroughly with hydrozone, one part to twelve parts of lukewarm water, and she returned home rejoicing, the turbinal wound being in good condition, healing nicely.

Next morning she came to my office for treatment, and stated she had enjoyed perfect freedom in breathing through that nostril until about four o'clock in the morning, when, changing her position in bed, that side became suddenly obstructed. After cleansing the nostril, which was seemingly full of an offensive discharge, I discovered this body, which was attached at the posterior end on the outer side of the inferior meatus, lying, as it were, in a groove or pocket.

The anterior or loose end of it was sharp like a spiculum of bone and black in color; it was freely movable about its long axis, so that you could pass a cotton-holder around it and lift it from its bed. After cocainizing, I grasped it with a dressing forceps, and, giving it a twist, removed it. I then thoroughly cleansed and disinfected the cavity with the hydrozone solution, which removed the odor and rendered the cavity wholesome.

The next day the two smaller pieces were removed while cleansing and treating the nose. They were loose, and seemed as though they had just scaled off from the bed where the larger piece had lain.

The spraying of the nasal cavity with hydrozone, followed by the use of glycozone, constituted the treatment for the next four days, by which time the offensive odor had entirely disappeared, and the parts had assumed a healthy condition.

This concretion formed on the outer side of the inferior meatus, and as it grew larger it obstructed the flow of tears through the nasolachrymal canal, as evidenced by the overflow of tears from the left eye, which condition ceased immediately after removal of the rhinolith.

The secondary hemorrhage was evidently due to a relaxation of the pressure on the vessels of the turbinate, owing to the calculus being disturbed in its position when the patient was retching.

As to the exciting cause of the formation in the case of this young lady, I could get only a negative history, there being no recollection of any foreign object having been put up the nose in her childhood. Being desirous of ascertaining, if possible, what served as a nucleus, and at the same time of finding out the composition of the formation, I cut it in two.

Microscopical examination reveals that it is composed of amorphous phosphates, undoubtedly the phosphates of calcium and sodium, which came from the tears.

There has been a marked improvement in the young lady's condition since the removal of the rhinolith; overflowing of the tears in the left eye has ceased, nasal respiration has become perfect, her voice has lost the nasal twang, and her general health has improved rapidly, as indicated by the fact that she has gained four pounds in weight since the operation (four weeks ago), and is still improving.—*New York Medical Journal*, July 8th, 1898.

